# Comparison of unilateral biportal endoscopic discectomy versus percutaneous endoscopic lumbar discectomy for the treatment of lumbar disc herniation: A systematic review and meta-analysis

**DOI:** 10.1097/MD.0000000000030612

**Published:** 2022-09-30

**Authors:** Xu Ma, Wenyi Li, Shangju Gao, Can Cao, Chuntao Li, Liang He, Meng Li

**Affiliations:** a Hebei North University, Zhangjiakou, Hebei, China; b Spinal Surgery Medical Team of Orthopedics, Hebei General Hospital, Shijiazhuang, Hebei, China; c Hebei Medical University, Shijiazhuang, Hebei, China.

**Keywords:** lumbar disc herniation, minimally invasive surgery, percutaneous endoscopic lumbar discectomy, treatment outcome, unilateral biportal endoscopic discectomy

## Abstract

The purpose of this study was to analyze unilateral biportal endoscopic discectomy (UBE) and percutaneous endoscopic lumbar discectomy (PELD) for the treatment of lumbar disc herniation.

**Methods:**

PubMed, EMBASE, Web of Science, Cochrane Database, CNKI, and Wanfang databases were searched online. All statistical analyses were performed using STATA 16.0.

**Results:**

The selection criteria were met by 6 studies with a total of 281 patients (142 cases in the UBE group and 139 cases in the PELD group) and good methodological quality. PELD has the potential to improve outcomes such as operation time and intraoperative hemorrhage (MD = 36.808, 95% CI (23.766, 49.850), *P* = .000; MD = 59.269, 95% CI (21.527, 97.010), *P* = .000) compared with UBE. No differences were found in the back pain VAS score at preoperative (MD = −0.024, 95% CI [−0.572, 0.092], *P* = .998), at 1 day after operation (MD = −0.300, 95% CI [−0.845, 0.246], *P* = .878), the VAS score of leg pain at preoperative (MD = −0.099, 95% CI [−0.417, 0.220], *P* = .762), at 1 day after operation (MD = 0.843, 95% CI [0.193, 1.492], *P* = .420), at 1 month after operation (MD = −0.027, 95% CI [−0.433, 0.380], *P* = .386), at 6 months after operation (MD = 0.122, 95% CI [−0.035, 0.278], *P* = .946), hospital stay (MD = 3.708, 95% CI [3.202, 4.214], *P* = .000) and other clinical effects between UBE and PELD group.

**Conclusions:**

There are no significant differences in clinical efficacy between UBE and PELD, according to the research. However, PELD has the potential to improve outcomes such as operation time and intraoperative hemorrhage. As just a result, PELD is better suited in the treatment of lumbar disc herniation.

## 1. Introduction

Lumbar disc herniation (LDH), which is the most common cause of back pain and sciatica, is 1 of the most significant health disorders with a high medical treatment cost.^[1]^ The prevalence rate is rising with the passage of time, and it is showing a younger tendency.^[2]^ In most cases, conservative treatment achieves an acceptable result. Surgery appears to be unavoidable for those who have failed to respond to conservative treatment. A variety of minimally invasive discectomy procedures have been developed as a result of the development of minimally invasive surgery.^[3]^ Kambin introduced percutaneous endoscopic lumbar discectomy (PELD) as a less invasive spine surgical option in the late 1980s.^[4]^ PELD has been widely used for LDH as a spinal minimally invasive surgery. It has successful outcomes compared to conventional open or microendoscopic surgery.^[5,6]^

In recent years, unilateral biportal endoscopic discectomy (UBE) is a rapidly growing surgical method that uses arthroscopic system for treatment of LDH. The technique has 2 independent channel endoscopies, which provide a clear and magnified surgical field that improves operational flexibility. UBE can be an effective treatment modality for LDH. The anatomic path and endoscopic image are comparable to those of a traditional discectomy. UBE has a sufficient and direct fragmentectomy and discectomy, which results in the same clinical outcomes as open microdiscectomy. UBE was thought to provide a viable alternative to traditional microscopic surgery.^[7]^

Compared with conventional microscopic operation, PELD and UBE have better curative effect for LDH. Therefore, as 2 kinds of minimally invasive surgery, which is better in the treatment of LDH is a clinical problem. However, at present, there is no systematic review and evaluation report on the PELD and UBE for LDH. We carried out this systematic review and meta-analysis to determine the priority of UBE and PELD for the treatment of LDH.

## 2. Materials and methods

### 2.1. Study selection and search strategy

A comprehensive search was performed in PubMed, EMBASE, Web of Science, Cochrane database, CNKI, and Wanfang databases were used for identifying relevant studies since the date of inception to March 2022. We also searched trial registries of ongoing trials. When the criteria for inclusion or exclusion of a study were controversial, the corresponding author was consulted. The search strategy followed the identification and screening guidelines established by PRISMA statement.^[8]^ The search strategy consisted of key words and commonly used synonyms and abbreviations including (“percutaneous endoscopic lumbar discectomy, PELD”, “transforaminal percutaneous endoscopic discectomy”, “percutaneous endoscopic transforaminal discectomy, PETD”, “percutaneous endoscopic interlaminar discectomy, PEID”, “unilateral biportal endoscopic, UBE”, “biportal endoscopic spinal surgery, BESS”, “irrigation endoscopic discectomy”, “twoportal endoscopic spinal surgery”, “translaminar lumbar epidural endoscopy”, “lumbar disc herniation”.) These terms were used in different Boolean combinations. To find more studies, we looked at the references listed in the eligible papers and relevant reviews.

### 2.2. Inclusion criteria

We included the following studies from the meta-analysis:

1. Study design: compared UBE with PELD for the treatment of LDH;
2. Single-level LDH with sciatica;
3. The technique of PELD could be percutaneous endoscopic transforaminal discectomy, PETD or percutaneous endoscopic interlaminar discectomy, PEID;
4. The study reported at least 1 desirable outcome.

Randomized controlled trials (RCTs) and retrospective or prospective cohort or case-control studies were among the study designs.

### 2.3. Exclusion criteria

Studies were excluded if they met the following criteria:

1. Non-contrastive study.
2. Studies that included patients spine abnormalities such as instability, spondylolisthesis, spinal stenosis, infection, tuberculosis, tumor, and so on.
3. Duplicate studies; meta-analysis; review article; case report; conference paper.
4. Studies involving >1 level segmental intervertebral disc herniation and re-operations.

### 2.4. Data extraction and quality assessment

The relevant data from the included studies was retrieved by 2 reviewers separately. When conflicts arise, the third step is required. The following data was extracted: basic study and population characteristics: first author, publication year, country of origin, study design, number of UBE and PELD groups, gender, age, and duration of follow-up; preoperative and postoperative clinical outcomes: back pain visual analogue score (VAS), leg pain VAS, Oswestry Disability Index (ODI) score, excellent and good ratio according to modified Macnab criteria; occurrence of complications. The Newcastle-Ottawa Scale (NOS) was used to evaluate the methodological quality of the studies (Table 1). This scale has 3 sections: ① selection (score: 3 points), ② comparability (score: 2 points), and ③ outcome (score: 3 points). The highest score for each study was 9, and a score between 5 and 9 was considered to be a lower risk of bias, while a score below 5 was considered to be a higher risk of bias.

**Table 1:** The Newcastle-Ottawa Scale (NOS) of the included studies.

Study (author, yr)	Selection	Comparability	Exposure	Total quality score
Jiang, 2022	3	1	2	6
Hao, 2022	3	1	3	7
Gu, 2021	2	1	3	6
Zhu, 2021	2	1	2	5
Merter, 2020	3	2	1	6
Choi, 2018	3	2	2	7

### 2.5. Data synthesis and statistical analysis

STATA MP 16.0 was used to conduct the meta-analysis (Stata Corp LLC, College Station, TX). Continuous data were calculated by mean differences (MD) with 95% confidence intervals (CIs), and dichotomous data were calculated by odd ratio (OR) with 95% CI. The chi-squared test and the degree of inconsistency (*I*^2^) were used to evaluate heterogeneity. When “*I*² < 50%” and “*P* > .1” showed a high degree of between-study heterogeneity. A random-effect model was used if heterogeneity was observed (*I*²>50% or *P* < .1). Otherwise, the fixed-effect model was used. *P* < .05 was regarded as statistically significant. Funnel plots were used to analyze potential publication bias.

### 2.6. Ethics approval statement

This study does not need to be approved by moral and ethical clerks.

## 3. Results

### 3.1. Search results

**Figure 1.**
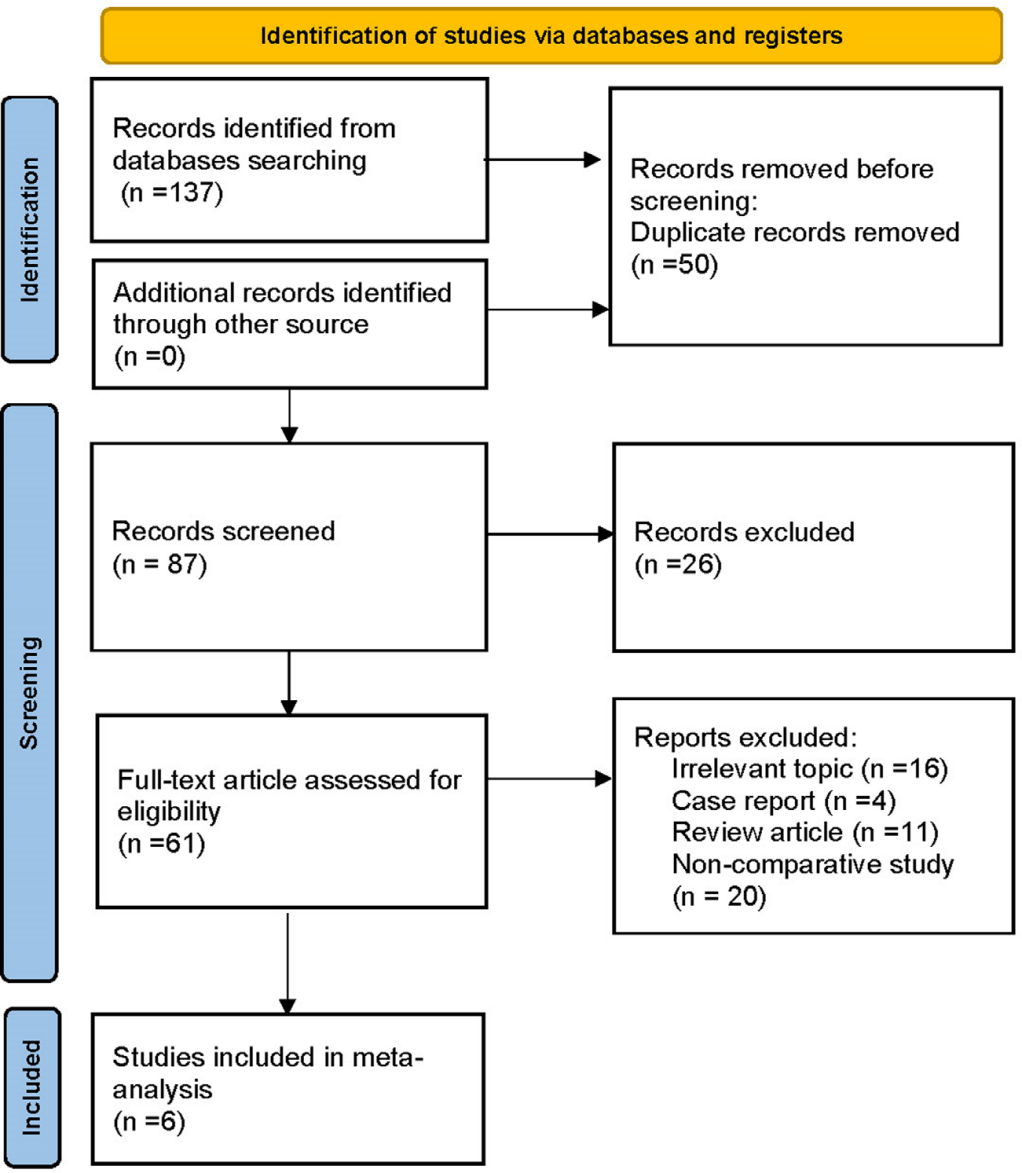
Flow diagram of the study identification and selection process.

The detailed results of the search for relevant literature based on the strategy described above was shown in Figure 1. A total of 137 articles were identified. Ultimately, 6 articles that enrolled 281 patients met the inclusion criteria.

### 3.2. Study characteristics and quality assessment

A total of 6 articles^[9–15]^ that included 281 patients (142 cases for UBE group and 139 cases for PELD group) met the inclusion criteria. The concrete characteristics of the included studies were summarized in Table 2. All studies were non-randomized control. The quality of trials was assessed by Newcastle–Ottawa scale (Table 1). The highest score for each study was ≥5 points. It can be considered that the overall quality of the literature included in the study is high.

**Table 2:** Baseline characteristics of the studies included in the meta-analysis.

Study (author, yr)	Design	Country	Number of patients (male/female)		Patient age (yr)		Follow-up time (mo)		Outcomes
			UBE	PELD	UBE	PELD	UBE	PELD	
Jiang, 2022	Retrospective study	China	13/17	10/14	46.25 ± 12.78	46.10 ± 10.45	6.40 ± 0.29	6.36 ± 0.21	①②③⑤⑥⑦⑧⑨
Hao, 2022	Retrospective study	China	14/6	8/12	58.2 ± 10.2	59.3 ± 7.8	At least 6 mo	At least 6 mo	①②③⑤⑥⑦⑨
Gu, 2021	Retrospective study	China	32	32	–	–	At least 6 mo	At least 6 mo	③④⑥⑧⑨
Zhu, 2021	Retrospective study	China	7/8	11/7	54 (38–78)	56 (25–79)	6–18	6–18	③④⑥⑧⑨
Merter, 2020	Multicenter prospective cohort study	Turkey and Japan	14/11	16/9	46.04	44.76	–	–	③
Choi, 2018	Prospective observational study	Korea	10/10	11/9	46.00 ± 8.91	42.90 ± 6.53	–	–	①②③⑤⑦⑧

① Back pain visual analogue score (VAS); ② Leg pain visual analogue score (VAS); ③ Operation time; ④ The number of intraoperative fluoroscopy; ⑤ Intraoperative hemorrhage; ⑥ Oswestry Disability Index; ⑦ Clinically satisfactory; ⑧ Hospital stay; ⑨ Complications.

PELD = percutaneous endoscopic lumbar discectomy, UBE = unilateral biportal endoscopic discectomy.

### 3.3. Results of meta-analysis

#### 3.3.1. Back pain visual analogue score (VAS).

**Figure 2.**
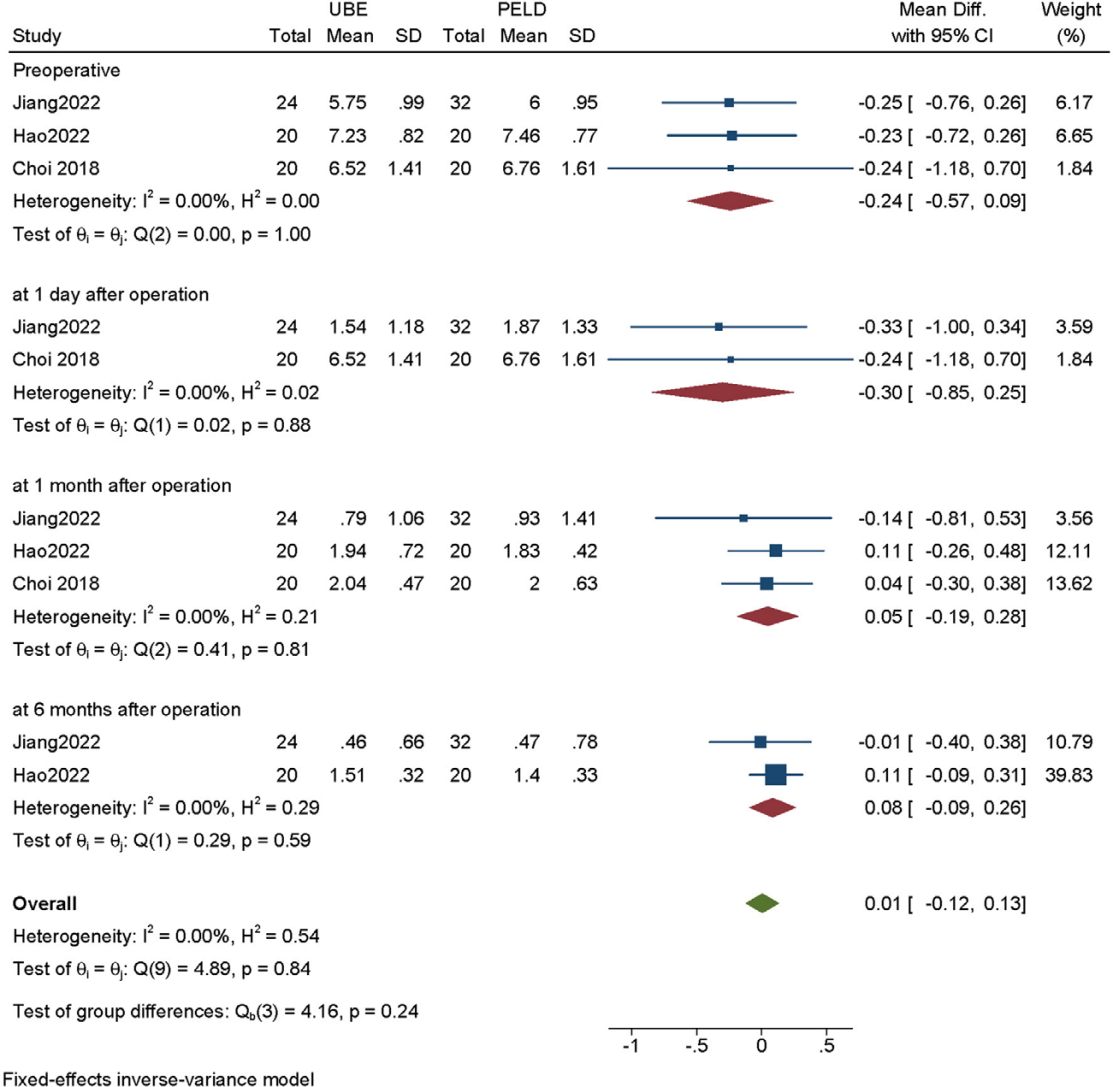
Forest plot for back pain visual analogue score at preoperative, at 1 day after operation, at 1 month after operation, at 6 months after operation between UBE and PELD. PELD = percutaneous endoscopic lumbar discectomy, UBE = unilateral biportal endoscopic discectomy.

The VAS score of back and pain was available from 3 studies.^[9,10,15]^ Heterogeneity among the studies was small (*I*^2^ = 0%), so the fixed effect model was used for meta-analysis. According to the different follow-up time, we conducted a subgroup analysis. There are no significant difference in VAS score of back pain between UBE and PELD group at preoperative (MD = −0.024, 95% CI [−0.572, 0.092], *P* = .998), at 1 day after operation (MD = −0.300, 95% CI [−0.845, 0.246], *P* = .878), at 1 month after operation (MD = 0.047, 95% CI [−0.188, 0.282], *P* = .814), at 6 months after operation (MD = 0.084, 95% CI [−0.094, 0.263], *P* = .590) (Fig. 2).

#### 3.3.2. Leg pain visual analogue score (VAS).

**Figure 3.**
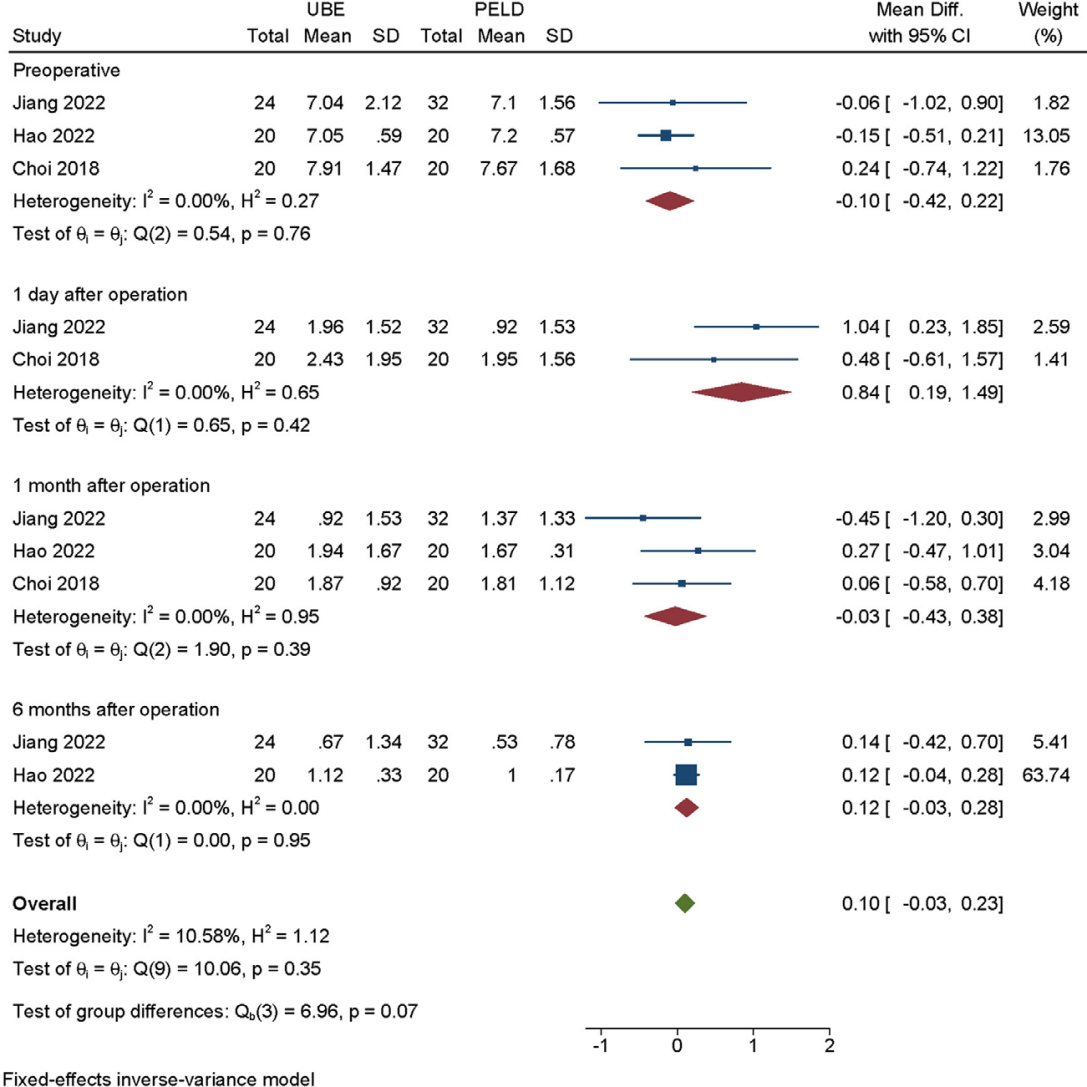
Forest plot for leg pain visual analogue score at preoperative, at 1 day after operation, at 1 month after operation, at 6 months after operation between UBE and PELD. PELD = percutaneous endoscopic lumbar discectomy, UBE = unilateral biportal endoscopic discectomy.

The VAS score of leg pain was available from 3 studies.^[9,10,15]^ Analysis indicated that there was high heterogeneity among the studies (*I*^2^ = 10.58%) and a fixed effect model was used. Subgroup analysis showed significant difference in VAS score of back pain between UBE and PELD groups at preoperative (MD = −0.099, 95% CI [−0.417, 0.220], *P* = .762), at 1 day after operation (MD = 0.843, 95% CI [0.193, 1.492], *P* = .420), at 1 month after operation (MD = −0.027, 95% CI [−0.433, 0.380], *P* = .386), at 6 months after operation (MD = 0.122, 95% CI [−0.035, 0.278], *P* = .946) (Fig. 3).

#### 3.3.3. Operation time.

**Figure 4.**
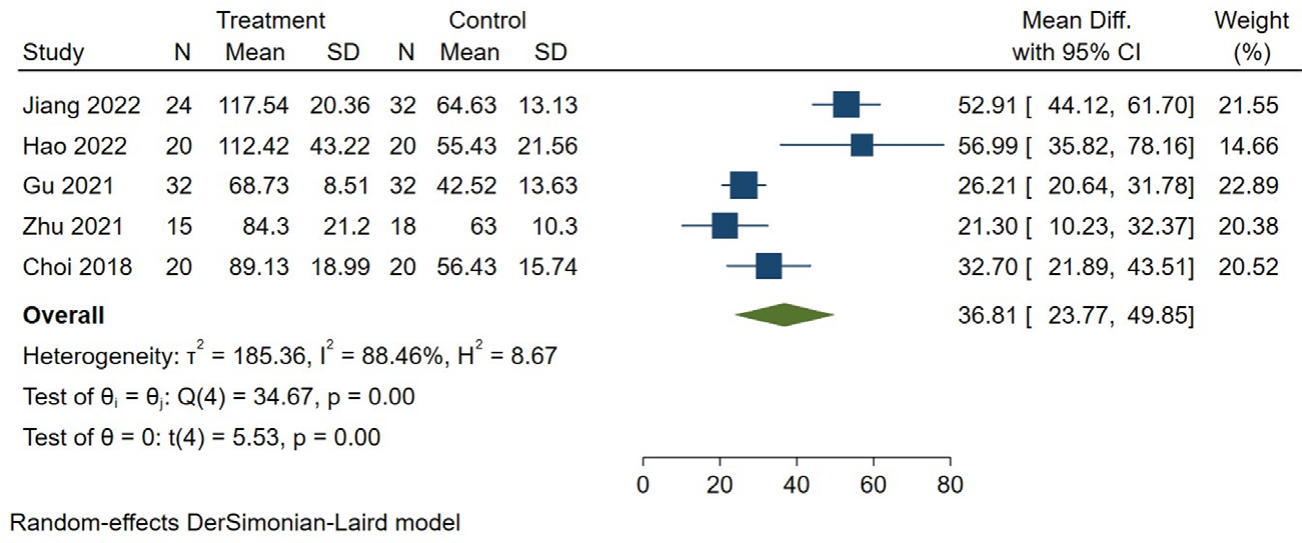
Forest plot for operation time between UBE and PELD. PELD = percutaneous endoscopic lumbar discectomy, UBE = unilateral biportal endoscopic discectomy.

The operation time was available from 6 studies.^[9–12,14,15]^ The heterogeneity was high (*I*^2^ = 96.42%). After sensitivity analysis of the study, it was found that Merter et al^[14]^ was the source of heterogeneity. The main reason was that it was a multi-center study and the heterogeneity was large due to the difference of surgeons, so we decided to exclude literatures and continue the meta-analysis. The results showed that the operation time of UBE group was longer than PELD group (MD = 36.808, 95% CI [23.766, 49.850], *P* = .000) (Fig. 4).

#### 3.3.4. Intraoperative hemorrhage.

**Figure 5.**
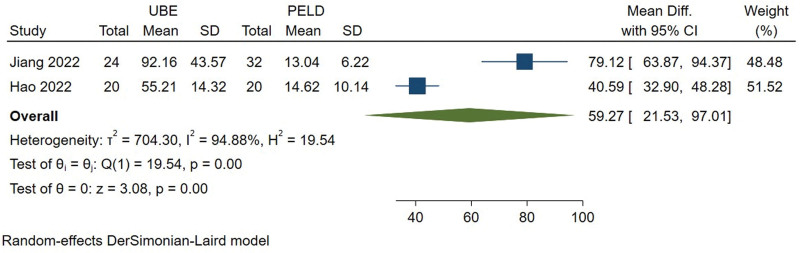
Forest plot for intraoperative hemorrhage between UBE and PELD. PELD = percutaneous endoscopic lumbar discectomy, UBE = unilateral biportal endoscopic discectomy.

The intraoperative hemorrhage was available from 2 studies.^[9,10]^ Meta-analysis indicated that there was high heterogeneity among the studies (*I*^2^ = 94.88%) and a random effect model was used. The results showed significant difference (MD = 59.269, 95% CI [21.527, 97.010], *P* = .000) between UBE and PELD group at intraoperative hemorrhage (Fig. 5).

#### 3.3.5. The number of intraoperative fluoroscopy.

**Figure 6.**
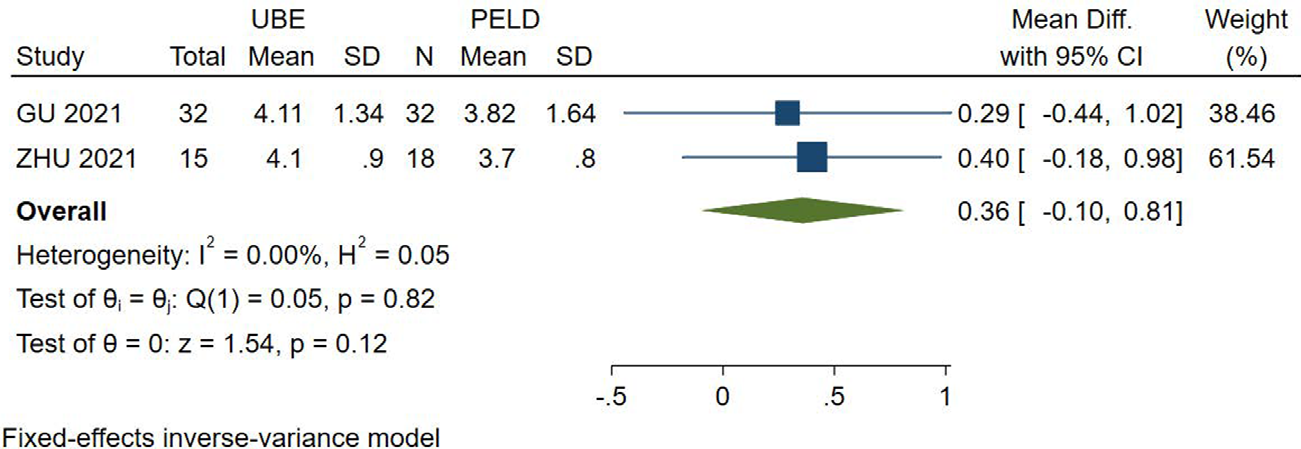
Forest plot for the number of intraoperative fluoroscopy between UBE and PELD. PELD = percutaneous endoscopic lumbar discectomy, UBE = unilateral biportal endoscopic discectomy.

The number of intraoperative fluoroscopy was available from 2 studies.^[11,12]^ Analysis indicated that there was high heterogeneity among the studies (*I*^2^ = 0.00%) and fixed effect model was used. There are no significant difference (MD = 0.358, 95% CI [−0.097, 0.813], *P* = .123) between UBE and PELD group at the number of intraoperative fluoroscopy (Fig. 6).

#### 3.3.6. Oswestry Disability Index (ODI) score.

**Figure 7.**
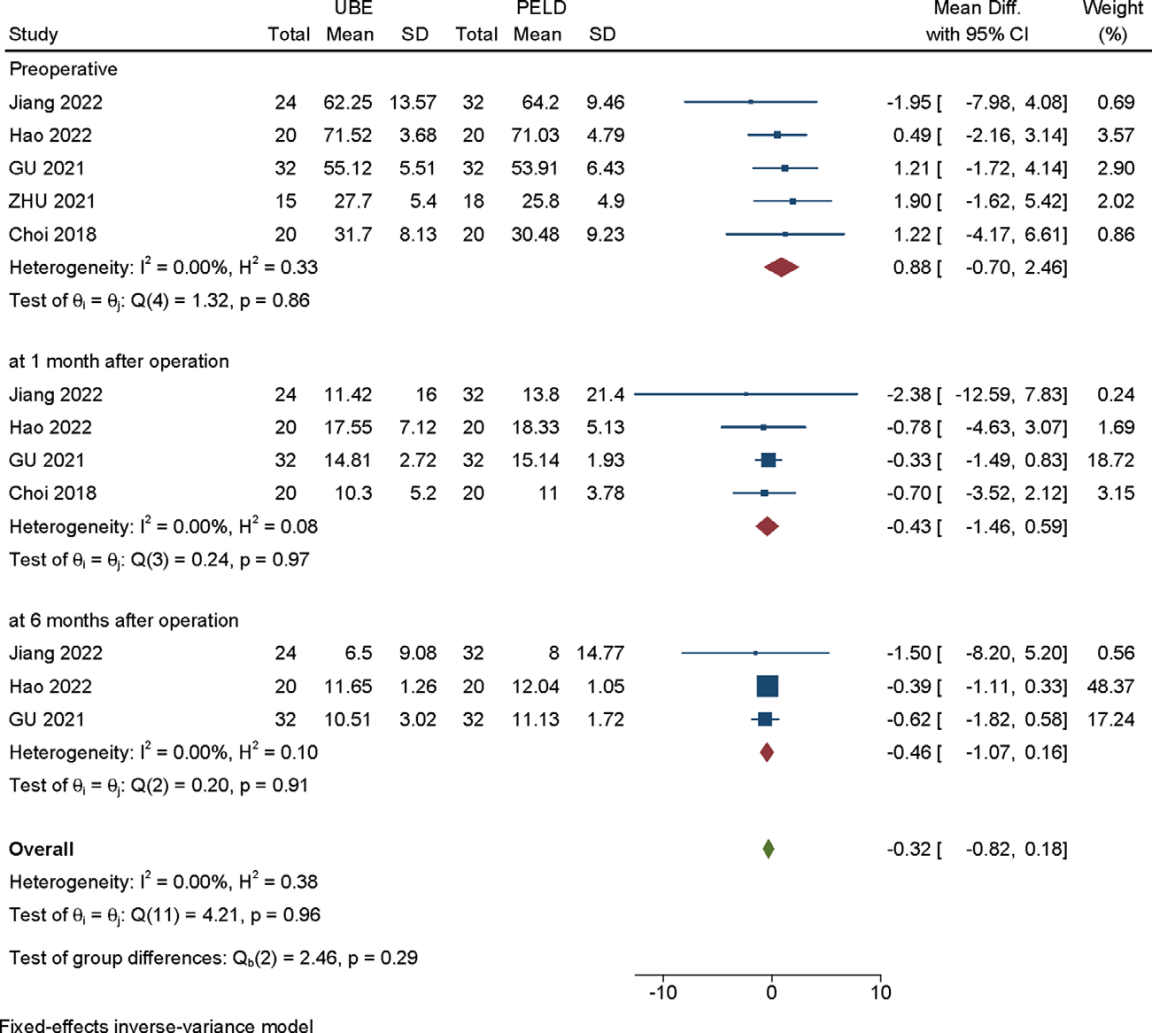
Forest plot of subgroup analysis for the ODI score between UBE and PELD at different follow-up time. ODI = Oswestry Disability Index, PELD = percutaneous endoscopic lumbar discectomy, UBE = unilateral biportal endoscopic discectomy.

The ODI score was available from 5 studies.^[9–12,15]^ Heterogeneity among the studies was small (*I*^2^ = 0%), so the fixed effect model was used for meta-analysis. According to the different follow-up time, we conducted a subgroup analysis. There are no significant difference in ODI score between UBE and PELD groups at preoperative (MD = 0.878, 95% CI [−0.700, 2.455], *P* = .859), at 1 month after operation (MD = −0.432, 95% CI [−1.456, 0.593], *P* = .814), at 6 months after operation (MD = −0.459, 95% CI [−1.074, 0.155], *P* = .906) (Fig. 7).

#### 3.3.7. Modified MacNab evaluation (excellent or good).

**Figure 8.**
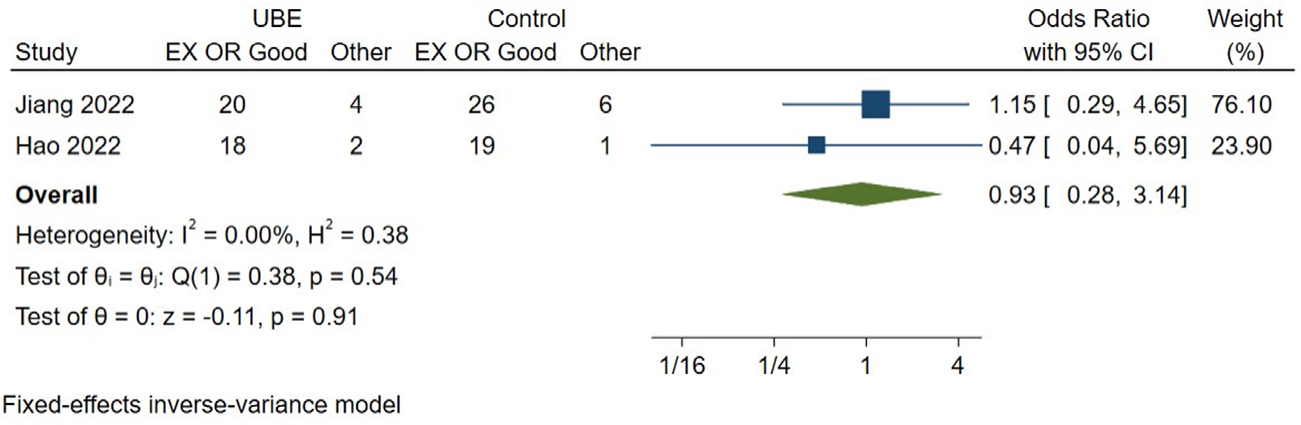
Forest plot for the excellent or good rate of Modified MacNab evaluation between UBE and PELD. PELD = percutaneous endoscopic lumbar discectomy, UBE = unilateral biportal endoscopic discectomy.

The excellent or good rate of Modified MacNab evaluation was available from 2 studies.^[9,10]^ Heterogeneity among the studies was small (*I*^2^ = 0%), so the fixed effect model was used for meta-analysis. There are no significant difference in excellent or good rate of Modified MacNab evaluation between UBE and PELD group (MD = 0.933, 95% CI [0.277, 3.144], *P* = .911) (Fig. 8).

#### 3.3.8. Hospital stay.

**Figure 9.**
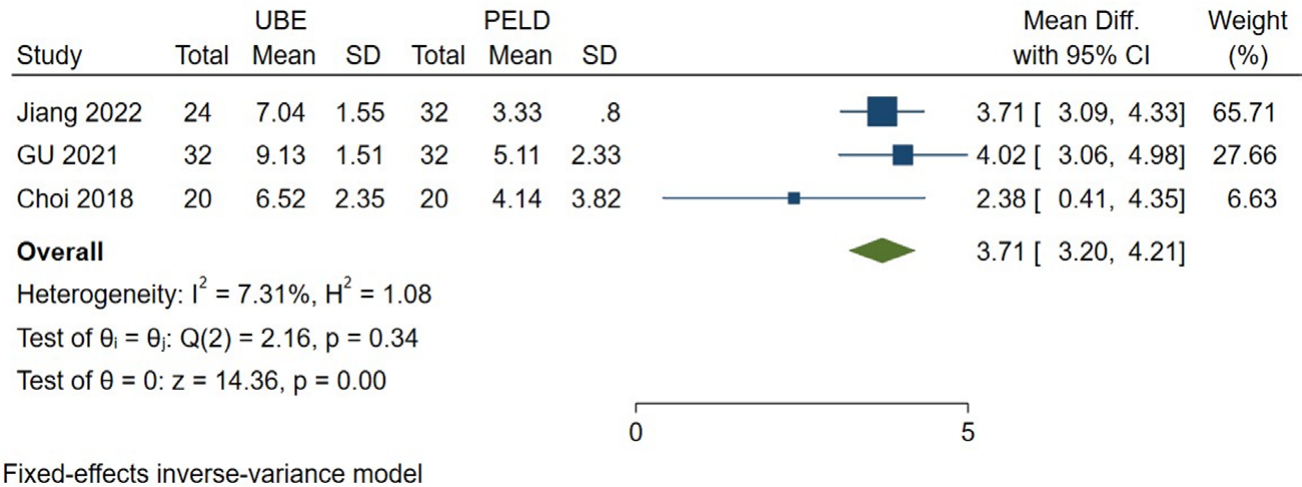
Forest plot for hospital stay between UBE and PELD. PELD = percutaneous endoscopic lumbar discectomy, UBE = unilateral biportal endoscopic discectomy.

The hospital stay was available from 4 studies.^[10–12,15]^ Heterogeneity among the studies was high (*I*^2^ = 92.81%). After sensitivity analysis of the study, it was found that Zhu^[11]^ was the source of heterogeneity. We decided to exclude literatures and continue the meta-analysis. When Zhu^[11]^ is removed, heterogeneity among the studies was small (*I*^2^ = 7.31%), so the fixed effect model was used for meta-analysis. The results showed significant difference (MD = 3.708, 95% CI [3.202, 4.214], *P* = .000) between UBE and PELD group at hospital stay (Fig. 9).

#### 3.3.9. Incidence of complications.

**Figure 10.**
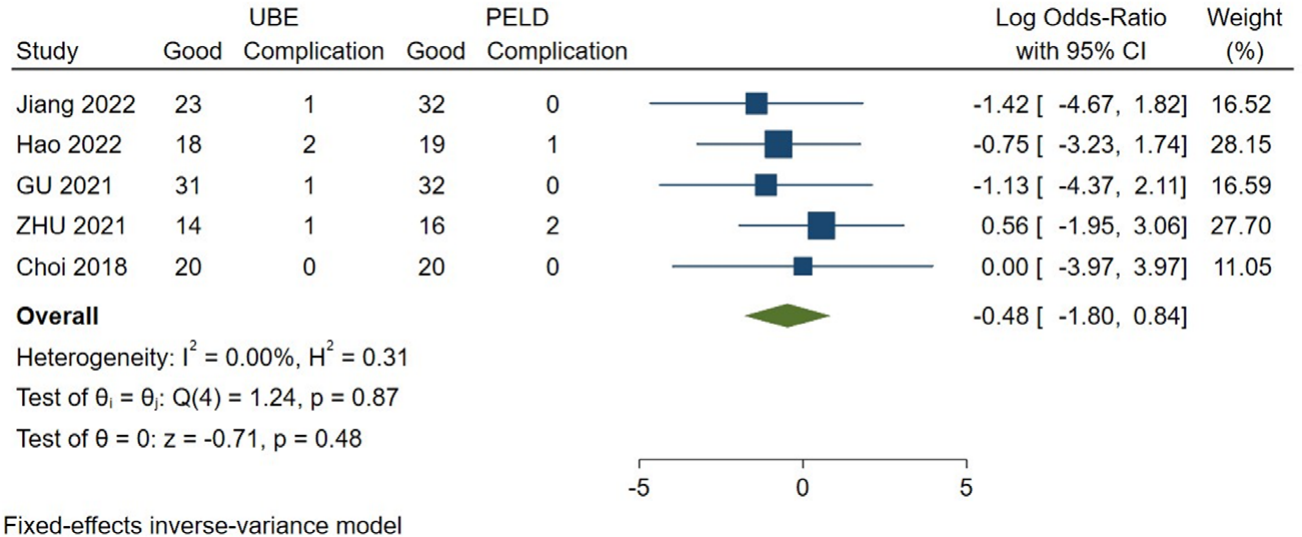
Forest plot for the incidence of complications between UBE and PELD. PELD = percutaneous endoscopic lumbar discectomy, UBE = unilateral biportal endoscopic discectomy.

The incidence of complications was available from 5 studies.^[9–12,15]^ Heterogeneity among the studies was small (*I*^2^ = 0%), so the fixed effect model was used for meta-analysis. There are no significant difference in the incidence of complications between UBE and PELD group (MD = 0.620, 95% CI [0.166, 2.318], *P* = .871) (Fig. 10).

## 4. Discussion

Since PELD was invented, it has experienced many innovations with major breakthroughs. It is these innovations that make PELD more and more widely used. In 1997, Yeung and Tsou^[16]^ developed the Yeung endoscopic spine system (YESS) and Hoogland et al^[17]^ developed the transforaminal endoscopic spine system (TESSYS) techniques in 2003, these 2 technologies make up percutaneous endoscopic transforaminal discectomy (PETD). Afterwards, Choi et al^[18]^ introduced percutaneous endoscopic interlaminar discectomy (PEID) in 2006. With the development of the enhanced recovery after surgery, minimally invasive spine surgery technology has increased interest of surgeon. PELD results in favorable long-term outcomes, has been standardized as a representative minimally invasive spine surgical technique for LDH treatment.^[19,20]^ However, PELD has a steep learning curve.^[21]^ Serious complications such as dural injury and nerve root injury will occur once the operation is wrong.^[22]^ About the problem of the steep learning curve of PELD, Wang et al^[23]^ recommend that extensive conventional open surgery experience and training of minimally invasive spine surgery such as demonstration teaching by experienced minimally invasive spine surgeon before attempting the PELD technique.

UBE was a new method that combined the advantages of interlaminar endoscopy and microscopic surgery. The UBE system used independent channels for instruments. The combination of observation channel and operation channel enables the instrument to move more widely, thus obtaining a better range of decompression and exploration. The endoscope used in biportal surgery is the same as those used in knee arthroscopy. Therefore, the endoscopy instruments can be shared with sports medicine surgeons for knee or shoulder arthroscopy; this can reduce medical costs.

In previous study, compared with conventional open discectomy, both UBE and PELD have been considered great alternatives for LDH. The reason was it has more advantages just like protection of back muscles and bony structures, shorter hospitalization, reduced intraoperative hemorrhage, and faster recovery.^[5,7,24]^ The number of reports on UBE has been increasing in recent years. UBE has been paid more and more attention by spine surgeons and has caused extensive study and discussion.

Compared with PELD technology, UBE technology has its own unique advantages. First of all, the 2 channels used by UBE, compared with PELD, are not restricted by pipes, the operation is more flexible, and the angle can be adjusted arbitrarily. UBE seemed to have a relatively short learning curve period. There are research reports^[25]^ that the overall complication rate in early learning period was 10.3%. The operation of UBE is similar to that of open discectomy, spinal surgeons are more familiar with it, and the learning curve is smoother than PELD technique. However, for the treatment of LDH, the choice of UBE or PELD is still inconclusive.

Through meta-analysis, we found that compared with UBE, PELD showed superiority in operation time and intraoperative hemorrhage compared with UBE, which supported the advantages of PELD in less invasion and enhanced recovery. This is consistent with the published research at present.^[9,10]^ In addition, at the previous studies, PELD was associated with various advantages relative to UBE, including shorter hospital stays, lower ODI score at 3 days after operation and less total hospitalization costs. Because of the small sample size and high heterogeneity in our meta-analysis, there are no difference was found in our meta-analysis. These findings may indicate that PELD has a minimal tissue injury after the procedure compared with UBE.

About the tissue injury, Choi et al^[15]^ conducted a study using Creatine Phosphokinase (CPK) and C-reactive protein (CRP) as indicators to evaluate the injury of the paraspinal muscle during surgery. This study showed that the PELD group had significantly lower CPK and CRP levels of the high-intensity lesion in the paraspinal muscle than the UBED and Microdiscectomy groups. Shin et al^[26]^ reported that the CPK level 3 days postoperatively was higher than the preoperative CPK. A strong relationship between postoperative elevation in CPK levels and surgical invasiveness has been shown. PELD use a muscle-splitting technique with sequential dilators and blunt obturator to preserve the integrity of the paraspinal muscle. UBE combines muscle-splitting and, to a small extent, muscle-stripping techniques. The shortcomings of UBE compared with PELD are that UBE needs 2 incisions, the observation channel is about 0.5 cm, and the operation channel is around 1 cm.^[27]^ UBE usually requires general anesthesia, so the cost of UBE is relatively high.^[28]^ The above factors may be the reasons for the long operation time, more tissue injury, and higher postoperative ODI score of UBE. Therefore, PELD is more appropriate in the treatment of LDH.

Hua et al^[29]^ have conducted a study of UBE and PELD in the treatment of lumbar spinal stenosis. The results found that UBE in the treatment of lumbar spinal stenosis and PELD achieved similar results, but in the operation time, the UBE group was shorter than the PELD group. UBE may be superior to PELD in the treatment of lumbar spinal stenosis. Of course, this needs to be further proved by a larger sample of research.

The following are some of the limitations of this meta-analysis. To begin, all nonrandomized and small sample studies were included; portions of the analysis had <3 studies and had a significant level of heterogeneity. Even when random-effect was applied, the test’s effectiveness may suffer. Furthermore, because not all articles specify the specific type of disc herniation, we were unable to conduct further subgroup analysis. This allows us to see if there are any changes in the treatment of different forms of disc herniation between UBE and PELD. To strengthen this study, more well-defined RCTs with big samples are needed.

## 5. Conclusions

The evidence suggests that there are no significant differences in efficacy and safety between UBE and PELD. PELD has the potential to improve outcomes such as operation time and intraoperative hemorrhage. As just a result, PELD is better suited in the treatment of LDH. However, a more substantial body of evidence is required. More multicenter RCTs are required before achieving a definitive conclusion.

## Author contributions

**Conceptualization:** Wenyi Li.

**Data curation:** Xu Ma, Shangju Gao, Can Cao, Chuntao Li, Liang He, Meng Li.

**Investigation:** Xu Ma, Chuntao Li, Liang He, Meng Li.

**Methodology:** Xu Ma, Chuntao Li.

**Software:** Xu Ma.

**Supervision:** Wenyi Li.

**Visualization:** Xu Ma, Chuntao Li.

**Writing – original draft:** Xu Ma.

**Writing – review & editing:** Wenyi Li.
